# Lurbinectedin, a DNA minor groove inhibitor for neuroendocrine neoplasms beyond small cell lung cancer

**DOI:** 10.18632/oncoscience.579

**Published:** 2023-06-14

**Authors:** Deepak Bhamidipati, Vivek Subbiah

**Keywords:** lurbinectedin, neuroendocrine tumors, NET, small cell lung cancer

Neuroendocrine tumors (NETs) encompass a variety of neoplasms which display a wide spectrum of biologic behavior, ranging from the aggressive neuroendocrine carcinoma (NEC) to often indolent well-differentiated NETs. For well-differentiated NETs, somatostatin analogs (SSAs) are widely accepted as an effective frontline therapy for progressive or symptomatic disease; however, subsequent therapy options such as capecitabine/temozolomide, sunitinib, everolimus, and radionuclide therapy in selected cases are associated with variable response rates (typically less than 20%) and limited progression-free survival. NECs can respond to platinum-based chemotherapy, but responses are typically short lived. There is evidence to suggest that neuroendocrine neoplasms such as small-cell lung cancer (SCLC) and pancreatic NETs are responsive to DNA alkylators such as temozolomide [1, 2]. Recently, lurbinectedin a DNA minor groove inhibitor and marine derivative was shown to inhibit oncogenic transcription through binding to CG-rich sequences near the promoters of protein-coding genes to promote apoptosis and cell-death [3]. Encouraging results from a phase II basket study of lurbinectedin as a second-line treatment for patients with SCLC, which demonstrated a 35% response rate, resulted in the FDA-approval of lurbinectedin in pre-treated patients with SCLC [4]. Moreover, in a subset analysis lurbinectedin was shown to effective treatment for platinum-sensitive relapsed SCLC, especially in patients with chemotherapy-free interval (CTFI) ≥180 days with an objective response rate of over 60% [5]. It was shown to be active in *BRCA1/2* germline mutated breast cancer [6]. In addition, it is active in Ewing sarcoma another small round cell tumor of neuroendocrine origin [7]. This bolstered the hypothesis that lurbinectedin could demonstrate activity in additional malignancies of neuroendocrine origin.

In a cohort of the lurbinectedin basket study, Longo-Muñoz et al. evaluated the antitumor activity of lurbinectedin in a range of NETs, including tumors of gastrointestinal, lung, and adrenal origin [8]. A total of 31 previously treated patients were enrolled, with the majority of patients having platinum-refractory disease and a Ki67 >10%. While the median progression free survival was low at 1.4 months, 2 patients achieved a partial response, and an additional 7 patients achieved stable disease lasting greater than 4 months, resulting in a disease control rate of 29%. Notably, most of the patients with clinical benefit had a proliferation index >10%.

As with any therapeutic which demonstrates preliminary evidence of efficacy, the identification of specific biomarkers which can identify patients who are most likely to benefit from lurbinectedin is of utmost importance. Perhaps lessons can be learned from the robust responses observed SCLC, which often share molecular and genetic features with gastroenteropancreatic (GEP) NECs such as *TP53* inactivation and *RB1* loss [9]. Neuroendocrine neoplasms which share a similar genetic and transcriptomic profiles to SCLC, such as NECs, may more often recapitulate the responses seen with lurbinectedin, which leverages the reliance of tumor cells on transcriptional activation. The ongoing EMERGE-201 study (NCT05126433) assessing lurbinectedin monotherapy includes a cohort of patients with NECs to identify whether this subset of neuroendocrine neoplasms displays response rates and disease control more akin to the activity seen in SCLC.

Nevertheless, there are fundamental differences in genomic alterations and biologic behavior between SCLC and GEP-NECs, such as the increased preponderance of *KRAS* mutations in NECs arising from the intestine and pancreas, which offer potentially distinct therapeutic vulnerabilities in NECs originating from sites outside of the lung. For example, while 5-Fluorouracil and irinotecan based combinations are not typically administered for patients with SCLC, they demonstrate activity in a significant percentage of patients with platinum-refractory GEP- NECs (22%) [10]. This stresses the importance of recognizing the distinct phenotype of NECs originating from different sites, despite many biologic similarities.

The optimal clinical and genomic phenotype which predicts response to lurbinectedin in neuroendocrine tumors is unclear, emphasizing the need for robust translational efforts to identify patterns associated with response and resistance. Several ongoing trials hope to further elucidate the role of lurbinectedin in high-grade neuroendocrine neoplasms, such as a clinical trial investigating the combination of lurbinectedin with irinotecan, which has activity in GEP neuroendocrine neoplasms (NCT02611024). Additionally, lurbinectedin is being assessed in combination with berzosertib, an ATR inhibitor, in patients with high-grade neuroendocrine neoplasms based on compelling evidence of synergistic activity in preclinical SCLC models (NCT04802174) [11]. Results and biomarker analyses from these studies and others are eagerly awaited to clearly define the role of lurbinectedin for the management of neuroendocrine neoplasms beyond SCLC.
